# Status of Lactate Dehydrogenase, Neutrophil-lymphocyte Ratio, Mean Platelet Volume, and Platelet-lymphocyte Ratio in Bell’s Palsy

**DOI:** 10.7759/cureus.7432

**Published:** 2020-03-27

**Authors:** Deniz Baklacı, İsmail Guler, İhsan Kuzucu, Rauf Oğuzhan Kum, Müge Özcan

**Affiliations:** 1 Otolaryngology, Kahramankazan State Hospital, Ankara, TUR; 2 Otolaryngology, Medipol University School of Medicine, Ankara, TUR; 3 Otolaryngology, Aksaray University Faculty of Medicine, Aksaray, TUR; 4 Otolaryngology, Ministry of Health Ankara City Hospital, Ankara, TUR

**Keywords:** bell’s palsy, ldh, nlr, mpv, plr

## Abstract

Objective: The inflammatory and vascular disorders have been proposed in the pathogenesis of Bell’s palsy (BP). Several studies investigated the role of inflammation and ischemia in BP using white blood cell (WBC) count and its subtypes. Here, we aimed to investigate lactate dehydrogenase (LDH), neutrophil-lymphocyte ratio (NLR), mean platelet volume (MPV), and platelet-lymphocyte ratio (PLR) in BP.

Methods: The study was conducted on 76 patients with BP and 60 healthy controls. The comparison of LDH, NLR, MPV, and PLR was made between groups. The relationships between LDH, NLR, MPV, and PLR were also investigated.

Results: The mean LDH concentrations and NLR were significantly high in BP group than in control group (p < 0.01, p < 0.05, respectively). There was no significant difference between groups in MPV and PLR (p = 0.195, p = 0.263, respectively).

Conclusion: Our results support the diagnostic value of high LDH concentrations in BP patients. Further studies are needed to investigate the relationship between LDH and the severity and prognosis of BP.

## Introduction

Bell’s palsy (BP) can be defined as acute, idiopathic, and unilateral paralysis of the facial nerve without any associated diseases [1]. There is a debate about the pathogenesis of BP. The inflammatory, infectious, and vascular ischemic reactions have been proposed as critical etiologic factors for the development of BP. The inflammatory theory suggests that inflammation on the facial nerve leads to thickening of the nerve sheet and swollen nerve jams in fallopian canal, especially in the labyrinthine segment [1]. Viral infections have been proposed as etiologic factors in BP. Viral reactivations cause inflammation and edema of the facial nerve [1]. On the other hand, the ischemic theory suggests that disturbed circulation in the vasa nervorum by a thrombus or vasospasm leads to nerve injury [2].

White blood cell (WBC) count and its subtypes have been identified as important biomarkers in cardiovascular diseases [3]. In later studies, the neutrophil-lymphocyte ratio (NLR) has been proposed as a novel potential marker to determine systemic inflammation in cardiac and noncardiac disorders [4].

Mean platelet volume (MPV) is an indicator of platelet functions and shows the production rate of platelets. High MPV values are associated with ischemic vascular conditions like atherosclerosis and thromboembolism [5]. Platelet-lymphocyte ratio (PLR) has been proposed as a marker for ischemic disorders. Patients with increased PLR value were found be at high risk for several peripheral vascular and coronary artery diseases [6].

Lactate dehydrogenase (LDH) enzyme is found in all human tissues. This enzyme catalyzes the conversion of lactate to pyruvic acid and is released into the extracellular area during inflammation-mediated cellular injury [7]. In the past, LDH was used as an inflammatory biomarker in infectious diseases [7-8].

In the literature, several studies investigated NLR, MPV, and PLR in BP. As per our literature knowledge, this study is the first one that investigated serum LDH concentrations in BP. We also aimed to investigate the relationship of LDH with NLR, MPV, and PLR in BP.

## Materials and methods

This clinical study was conducted retrospectively in a tertiary referral center on patients diagnosed with BP between January 2013 and December 2016. The control group consisted of healthy individuals with no evidence of otologic or facial nerve pathology. The study protocol was approved by the local ethical committee of our center (decree no: E-18-1847). An informed consent was obtained from all participating subjects. The study was conducted in accordance with the principles of the Declaration of Helsinki. The informed consents that include blood tests, medical therapy, and radiologic imagination were obtained from all patients. In this study, we retrieved the following data from our hospital's medical records: demographic characteristic, LDH, MPV, platelet, neutrophil, and lymphocyte counts. The patients and individuals who had an acute systemic infection, lung infection, renal disorders, liver disease, malignancy, chronic obstructive respiratory disorders, obstructive sleep apnea disease, connective tissue disease, ulcerative colitis or Crohn’s disease, diabetes mellitus, hypertension, active smoking, or current ear disorder were excluded from the study. Blood samples were collected from patients before starting steroid medication. All the patients were treated with methylprednisolone starting with a dose of 1 mg/kg/day, with a gradual dose reduction, and discontinuation at two weeks. Routinely, all patients underwent audiologic examination and MRI.

Hematologic analysis and LDH measurement

The LDH measurements were conducted on the Olympus 2700 auto-analyzer using commercial Olympus kits by enzymatic spectrophotometric (UV) method. Complete blood counts of the participants were obtained using Sysmex XE-2100 device (Sysmex Corp, Kobe, Japan), and erythrocyte, leukocyte, neutrophil, lymphocyte, and thrombocyte counts were used to determine NLR and PLR. NLR was calculated as a simple ratio between the absolute neutrophil and the absolute lymphocyte counts while PLR between the absolute platelet and the absolute lymphocyte counts.

Statistical analysis

Statistical analysis was carried out by using IBM SPSS statistics version 20 (SPSS, Inc, Chicago, IL, US). Data were expressed as mean ± standard deviation (SD) and percentage (%). Chi-square test was used for comparison of categorical data. Normality of data was examined with the Kolmogorov-Smirnov test. The Wilcoxon and Mann-Whitney *U* tests were used for nonparametric variables while independent and paired sample *t*-tests were used for parametric variables. p < 0.05 was considered as statistically significant.

## Results

A total of 76 BP patients who met the criteria mentioned above were included in this study. The control group consisted of 60 healthy individuals. The BP group consisted of 37 males (48.6%) and 39 females (51.4%), and the control group included 28 males (46.7%) and 32 females (53.3%). The mean age was 39.76 ± 9.02 years in the BP group while 39.31 ± 8.16 years in the control group with no statistically remarkable age and gender difference between the groups (p = 0.697, p = 0.815, respectively) (Table 1). 

**Table 1:** Comparison of groups in terms of age, gender, LDH, and blood parameters. BP, Bell's palsy; MPV, mean platelet volume; LDH, lactate dehydrogenase; NLR, neutrophil-lymphocyte ratio; PLR, platelet-lymphocyte ratio; SD, standard deviation. ^a^Student’s *t-*test. ^b^Mann-Whitney *U* test, ^c^Fisher’s Exact test. *p < 0,05, **p < 0,01.

		BP (n = 76)	Control (n = 60)		p
		Mean ± SD	Mean ± SD		
Age (years)		39.76 ± 9.02	39.31 ± 8.16		0.697^a^
MPV (fl)		8.33 ± 1.21	8.15 ± 0.81		0.195
LDH (U/L)		242.05 ± 68.09	186.20 ± 28.72		0.001**^a^
Platelet (10^3^/U)		254.31 ± 85.36	241.81 ± 59.45		0.213
Neutrophil (10^3^/U)		6.09 ± 3.28	3.32 ± 0.55		0.001**^a^
Lymphocyte (10^3^/U)		2.01 ± 1.66	1.94 ± 0.86		0.465
NLR		3.13 ± 1.97	1.71 ± 0.63		0.001*^b^
PLR		126.00 ± 51.29	116.93 ± 67.46		0.263
		n (%)	n (%)		
Gender	Male	37 (48.6%)	28 (46.7%)	0.815^c^	
	Female	39 (51.4%)	32 (53.3%)		

The mean LDH concentration was 242.05 ± 68.09 (U/L) in BP group, remarkably higher than of the control group, which was 186.20 ± 28.72 (U/L) (p < 0.001) (Table 1). The mean NLR value in the BP group, which was 3.13 ± 1.97, was statistically higher than of the control group, which was 1.71 ± 0.63 (p < 0.001) (Table 1). The mean MPV was 8.33 ± 1.21 in the BP group and 8.15 ± 0.81 in control group with no statistically remarkable difference between the groups (p = 0.195). The mean PLR value was 126.00 ± 51.29 in the BP group and 116.93 ± 67.46 in the control group, revealing no statistically remarkable difference between the two groups (p = 0.263). The mean neutrophil count was 6.09 ± 3.28 in the BP group and 3.32 ± 0.55 in the control group, statistically higher in the BP group (p < 0.001). The mean lymphocyte count was 2.01 ± 1.66 in BP group and 1.94 ± 0.86 in control group with no statistically remarkable difference between the groups (p = 0.564). The mean platelet count was 254.31 ± 85.36 in BP group and 226.13 ± 58.02 in control group, indicating a statistically remarkable difference between the two groups (p = 0.005).

## Discussion

Up to date, no studies have investigated serum LDH concentrations in BP. In our study, we investigated LDH concentrations in addition to the hematologic biomarkers such as NLR, MPV, and PLR that were previously studied in BP. The key finding is that serum LDH concentrations were remarkably higher in BP patients than in the control group. We also found significantly higher NLR in BP patients than in control group. There was no significant difference between groups in MPV and PLR.

The most prevalent cause of unilateral peripheral facial paralysis is BP. The incidence of BP is about 1-4/10.000 with a lifetime risk of 1 in 60 [9]. The pathogenesis of BP is not precise yet. The inflammatory and autoimmune diseases, vascular thromboembolic and ischemic diseases, and viral infections have been proposed as etiologic factors in the development of BP [9]. BP is characterized by acute onset hemifacial muscle paralysis, epiphora, pain around the auricula, disturbed taste sensation, and hypersensitivity to sounds. To make the diagnosis of BP; brain and brainstem pathologies, otologic pathologies which may lead to paresis and paralysis must be excluded.

Several studies are indicating that cell-mediated inflammatory process may be a key factor in the pathogenesis of BP. Yılmaz et al. compared serum inflammatory cytokine levels of between the patients with BP and healthy individuals. The results of the study showed that the IL-6, IL-8, and TNF-a levels were higher in the BP group compared to the control group [10]. Therefore, an inflammatory process, secondary to viral infection may be one of the pathogenic factors in the development of BP. Kilicaslan et al. investigated the level of procalcitonin, a proinflammatory marker in BP patients, and found that procalcitonin was remarkably higher in BP patients than in control, and there was a strong negative correlation between procalcitonin levels and recovery rates [11].

The LDH enzyme is present in the cytoplasm of all living cells. This enzyme catalyzes the oxidation of lactate to pyruvate in the glycolytic reactions. There are five different forms of LDH, and different isoenzymes (LDH1-5) are found in various tissues [12]. It is released into the extracellular area due to inflammation-mediated cellular injury and increased concentrations of LDH assist clinicians to diagnose some diseases. For example, LDH1 is highly found in heart muscle tissue and it was used for the diagnosis of myocardial infarction [12]. LDH was used as an inflammatory biomarker in infectious conditions such as bacterial meningitis, pulmonary diseases, and arthritis [7-8]. During viral upper airway infections (VUAIs), increased LDH concentrations in nasopharyngeal secretions were shown to be associated with the risk of developing acute otitis media [13]. Juhn et al. showed that serum immunoglobulin levels and LDH concentrations were significantly high during purulent and secretory otitis media infection in rats [14]. In our study, serum LDH concentrations were considerably higher in BP patients than in controls, and this finding would be regarded as an indicator of inflammatory process during the development of BP. To our literature knowledge, the role of inflammation in BP has not been studied by using LDH.

Recently, WBC count and its subtypes have been found as important inflammatory biomarkers in BP. Bucak et al. found that NLR and neutrophil levels were higher in patients with BP compared to the control group. They also found that NLR was higher in inadequately recovered patients compared to adequately recovered patients [15]. Similarly, in another study authors found a positive and remarkable correlation between increased NLR and the grade of BP [16]. In our study, consistent with literature, we found significantly increased NLR values in BP patients.

The high PLR and MPV have been shown to be associated with vascular thromboembolic and ischemic diseases. In a study, PLR was observed to be higher in patients with BP compared to healthy individuals. But, they did not find any correlation between PLR and grade of BP [17]. In another study, MPV was found to be high in patients with BP. As a result of that study, the authors concluded that higher MPV values would be a predictor marker for the severity and prognosis of BP [18]. In our study, we did not find higher MPV and PLR values in BP patients than in those controls.

The limitation of the present study is that we do not have sufficient data about the association between LDH concentrations and the grade and prognosis of BP. We believe that further prospective studies may show us whether LDH concentrations reflect the severity of BP and assist in predicting the prognosis of BP.

## Conclusions

The present study is the first study to investigate the association between LDH, NLR, MPV, and PLR levels and BP. High LDH and NLR support the inflammatory hypothesis on the development of BP. LDH might be considered as a diagnostic inflammatory marker in BP. Further studies are necessary to investigate the relationship between LDH and the grade and prognosis of BP. 
